# Beyond the Brain: Circulating Tumor Cells as a Tool for Diagnosis and Monitoring

**DOI:** 10.1200/PO-25-01199

**Published:** 2026-07-23

**Authors:** Francesca Lessi, Mariangela Morelli, Alessandro Di Gangi, Sara Franceschi, Francesca Di Lorenzo, Chiara Sandro, Paolo Aretini, Aldo Pastore, Gianmarco Ferri, Michele Menicagli, Francesco Pasqualetti, Carlo Gambacciani, Federico Villanacci, Vanna Zucchi, Samanta Cupini, Anna Luisa Di Stefano, Orazio Santo Santonocito, Chiara Maria Mazzanti

**Affiliations:** ^1^ Section of Genomics and Transcriptomics, Fondazione Pisana per la Scienza, Pisa, Italy; ^2^ Department of Radiology and Radiotherapy, UOC Radiotherapy, Istituto Oncologico Veneto, Padova, Italy; ^3^ Department of Surgical, Oncological and Gastroenterological Sciences (DiSCOG), Università di Padova, Padova, Italy; ^4^ Division of Neurosurgery, Spedali Riuniti di Livorno—USL Toscana Nord-Ovest, Livorno, Italy; ^5^ Division of Pathology, Spedali Riuniti di Livorno-USL Toscana Nord-Ovest, Livorno, Italy; ^6^ Division of Oncology, Spedali Riuniti di Livorno-USL Toscana Nord-Ovest, Livorno, Italy

## Abstract

**PURPOSE:**

Glioblastoma (GBM) is an aggressive brain tumor characterized by extensive heterogeneity and inevitable recurrence. Although extracranial metastases are rare, circulating tumor cells (CTCs) are consistently detectable in patients with GBM, challenging the idea of GBM as a strictly localized disease and suggesting CTCs as a promising liquid biopsy tool.

**METHODS:**

We analyzed genomic and phenotypic features of bulk CTCs (BCTCs) and single CTCs in 27 patients with GBM. Whole-exome sequencing of BCTCs and matched tumors confirmed tumor origin and assessed shared mutational and copy number alteration (CNA) profiles. Single CTCs isolated with DEPArray NxT underwent low-pass whole-genome sequencing. CTCs counts were compared across primary GBM, recurrent GBM, healthy donors, and non-GBM conditions. Single-cell RNA-seq from primary GBM cell lines was used to validate observations.

**RESULTS:**

BCTCs showed genomic alterations overlapping with those of matched tumors. CTCs were significantly more abundant in primary GBM than in recurrent disease or healthy controls, enabling the definition of a diagnostic cutoff of 4 CTCs per 5 mL of blood. CTCs were absent in the small cohort of radionecrosis cases analyzed, suggesting a potential role for CTCs in distinguishing neoplastic from non-neoplastic lesions, although further validation in larger cohorts is required. Single-cell analysis revealed marked heterogeneity, including CTCs lacking detectable CNAs. Similar CNA-negative populations appeared in scRNA-seq data sets, suggesting possible origin from the tumor microenvironment rather than fully malignant clones.

**CONCLUSION:**

CTCs are tumor-derived and heterogeneous biomarkers with strong diagnostic potential in GBM. The identified cutoff improves distinction between GBM and non-neoplastic diseases, supporting CTCs as a useful liquid biopsy tool for detection and monitoring. Further studies should clarify the biologic roles of distinct CTCs subpopulations.

## INTRODUCTION

Characterized by a poor prognosis with a median overall survival (OS) of approximately 15 months, glioblastoma (GBM) is the most common primary malignant tumor of the CNS.^1^ A key feature of GBM is its significant molecular heterogeneity, which contributes to the challenges in effective treatment.^2^ Despite initial intervention, recurrence is almost universally observed, leading inevitably to patient death.^3^

**CONTEXT**

**Key Objective**
Can circulating tumor cells (CTCs) be reliably quantified and molecularly characterized to serve as a noninvasive liquid biopsy tool for glioblastoma and reflect the genomic heterogeneity of the primary tumor?
**Knowledge Generated**
A diagnostic threshold of 4 CTCs per 5 mL of blood achieved 80% sensitivity and 77.3% specificity in distinguishing primary glioblastoma from healthy controls. Whole-exome sequencing of bulk CTCs, performed without whole-genome amplification, confirmed tumor origin through shared somatic mutations with matched tissues. Single-cell analysis revealed marked genomic heterogeneity, including a subpopulation lacking detectable copy number alterations, suggesting that circulating cells capture diverse components of the tumor ecosystem.
**Relevance**
CTCs represent a promising noninvasive tool for glioblastoma diagnosis and monitoring. The defined cutoff and molecular validation support clinical applicability, while distinct CTC subpopulations provide insight into tumor biology and the tumor microenvironment.

Circulating tumor cells (CTCs) are generally considered key players in the metastatic process.^4,5^ Although, extracranial metastases are extremely rare in GBM,^6^ the presence of CTCs in patients with GBM is now well documented.^7-10^ CTCs effectively represent a liquid biopsy, fundamentally differing from other components like circulating tumor DNA or extracellular vesicles because they are intact, viable cells. This critical distinction makes them a powerful tool for rapid access to tumor information and tracking disease progression over time,^11,12^ enabling comprehensive molecular and phenotypic analysis at the single-cell level without requiring a guiding molecular marker.

The profound intratumoral and intertumoral heterogeneity of GBM presents a formidable oncologic challenge. Our previous work,^13^ has indicated that CTCs isolated from patients with GBM also reflect this high degree of heterogeneity. However, the precise extent and implications of this CTC heterogeneity remain underexplored.

In this study, the first objective was to confirm the tumor origin of CTCs by analyzing pooled (bulk) CTC populations (BCTCs), rather than single cells, to capture a broader and more representative view of the tumor's genetic heterogeneity and clinically relevant alterations. Subsequently, we quantified the CTCs in patients with GBM, assessing whether their presence or abundance correlated with tumor progression. Understanding the molecular features of GBM-derived CTCs may also shed light on their unique ability to detach from the primary tumor, cross the blood-brain barrier, and survive in the circulatory system. These properties could mark them as a particularly aggressive subpopulation, potentially sharing traits with the tumor cells responsible for therapeutic resistance and intracranial recurrence.

## METHODS

### Study Population

A total of 43 individuals (N = 43) were included in our study. This cohort comprised 27 patients (n = 27) with GBM, including 20 (n = 20) with primary GBM and 16 (n = 16) with recurrent tumors, were collected from individuals undergoing surgical resection at the Department of Neurosurgery, Ospedali Riuniti di Livorno-USL Toscana Nord Ovest, Italy. In addition, 10 (n = 10) healthy individuals were included as controls. Notably, for 7 patients (n = 7), samples from both the primary tumor and the recurrence were available. The cohort included 9 female patients (n = 9, 33%), with a mean age of 63 years (standard deviation, 13). All the tumors were GBM *IDH1* and *IDH2* wild type according to 2021 WHO classification,^14^ and *MGMT* promoter methylation was detected in 15 cases (n = 15, 55%). In addition to these patients, 6 nonhealthy cases (n = 6) were included in our cohort: two *IDH1* mutated-gliomas, two cases of radionecrosis, one lymphoma, and one recurrent case of pleomorphic xanthoastrocytoma. This study was conducted according to the guidelines of the Declaration of Helsinki and approved by the Ethics Committee of the University Hospital of Pisa (787/2015). All patients provided written informed consent under a protocol approved by the Ethics Committee. Patient clinical and molecular characteristics, as well as treatment and procedural details, are summarized in Table 1.

**TABLE 1.** Clinical and Molecular Characteristics of the Study Cohort (n = 27)

Patient	Sex	Age, Years	*MGMT* Status	Tumor Istotype	Gene Mutated	*IDH* Status	Primary-Recurrence	Surgical Outcome (GTR, PR)	Pre-CTC Blood Draw Treatments	Time Last Radiotherapy (for recurrences) in Months
GBM1	M	47	Unmethylated	GBM	*BRAF*	WT	Primary	Not available	None	None
GBM2	M	77	Methylated	GBM		WT	Recurrence	STR	RT-TMZ	71
GBM3	M	76	Methylated	GBM		WT	Primary tumor	GTR	None	None
GBM4	M	38	Methylated	GBM		WT	Primary tumor	GTR	None	None
GBM5	F	55	Unmethylated primary tumor methylated recurrence	GBM		WT	Primary and recurrence tumor	GTR for primary and not available for recurrence tumor	RT-TMZ for recurrence	28
GBM6	F	52	Unmethylated	GBM	*PIK3CA*	WT	Recurrence	GTR	RT-TMZ	2
GBM7	M	52	Unmethylated	GBM		WT	Recurrence	Not available	RT-TMZ	6
GBM8	M	82	Methylated	GBM		WT	Primary tumor	STR	None	None
GBM9	F	56	Unmethylated	GBM		WT	Recurrence	GTR	RT-TMZ	4
GBM10	F	36	Unmethylated	GBM		WT	Primary tumor	GTR	None	None
GBM11	M	52	Methylated	GBM		WT	Primary tumor	Not available	None	None
GBM12	M	57	Unmethylated	GBM	*BRAF*	WT	Recurrence	GTR	RT-TMZ	16
GBM13	M	74	Methylated	GBM	Del19q	WT	Primary tumor	STR	None	None
GBM14	F	68	Unmethylated	GBM	*PIK3CA*-del 1p	WT	Primary tumor	GTR	None	None
GBM15	M	75	Methylated	GBM		WT	Primary and recurrence tumor	GTR	RT-TMZ for recurrence	12
GBM16	M	49	Methylated	GBM	*EGFR*	WT	Primary tumor	GTR	None	None
GBM17	M	71	Methylated	GBM	Codel 1p-19q	WT	Primary and recurrence tumor	STR	RT-TMZ for recurrence	8
GBM18	M	75	Methylated	GBM	Del 1p	WT	Primary tumor	GTR	None	None
GBM19	F	74	Methylated	GBM		WT	Primary tumor	STR	None	None
GBM20	M	69	Unmethylated	GBM		WT	Primary and recurrence tumor	STR	RT-TMZ for recurrence	6
GBM21	M	77	Methylated	GBM	*PIK3CA*	WT	Primary and recurrences tumor	GTR	RT-TMZ for recurrence	7 (recurrence 1) 17 (recurrence 2)
GBM22	F	67	Methylated	GBM	*PIK3CA*	WT	Primary tumor	PR	None	None
GBM23	F	73	Unmethylated	GBM	Del 1p	WT	Primary and recurrence tumor	GTR	RT-TMZ for recurrence	9
GBM24	M	69	Unmethylated	GBM	Del 19q	WT	Primary tumor	GTR	None	None
GBM25	M	55	Unmethylated	GBM		WT	Recurrence	STR	RT-TMZ	2
GBM26	F	43	Methylated	GBM		WT	Primary tumor	GTR	None	None
GBM27	M	73	Methylated	GBM	Codel 1p-19q	WT	Recurrence	GTR	RT-TMZ	9

NOTE. Demographic data, MGMT promoter methylation status, genetic mutations, and IDH status are reported for each patient with GBM. The table also details the tumor recurrence status, surgical outcomes, and treatment history before CTCs blood draw. For all patients, blood collection for CTCs analysis was performed immediately before surgical intervention.

Abbreviations: CTC, circulating tumor cell; GBM, glioblastoma; GTR, gross total resection; PR, partial resection; RT-TMZ, radiotherapy-Temozolomide; STR, subtotal resection; WT, wild-type.

### Human Glioblastoma Tissues and Blood Collection

Five milliliters of peripheral blood were collected in EDTA tubes for CTC detection immediately before surgical incision and before any tumor manipulation. Blood samples were obtained at the time of surgery, regardless of whether patients underwent surgery for primary or recurrent disease. Peripheral blood mononuclear cells (PBMCs) were obtained from whole blood through density gradient centrifugation (Ficoll Paque GE17-1440-02, Sigma-Aldrich) as the standard procedure. The pellet obtained was frozen vital and stored at –140°C.

Fresh GBM tissues were obtained at the time of the surgery, collected in MACS Tissue Storage Solution (Miltenyi Biotec, Bergisch Gladbach, Germany), and maintained in a viable state. These tumor tissues were used for DNA extraction and the establishment of primary cell lines.

### CTC Enrichment, Identification, and Single-Cell Isolation

PBMCs were enriched for CTCs using the Parsortix Cell Separation System (Angle plc, Surrey, United Kingdom) with a 6.5-μm separation cassette, according to the manufacturer's instructions. For CTC enumeration, enriched cell suspensions were fixed and stained by immunofluorescence using glial fibrillary acidic protein (GFAP) as a tumor-associated marker, CD45 as a leukocyte exclusion marker, and Hoechst 33342 for nuclear staining. Single cells were subsequently isolated using the DEPArray NxT system (Menarini Silicon Biosystems, Bologna, Italy) based on fluorescence labeling and morphology. CTC counts were normalized to the analyzed blood volume (5 mL). Detailed staining, sorting, and processing procedures are provided in the Data Supplement.

### WES of Bulk CTC-Enriched Fractions

WES was performed on primary tumor tissue, matched recurrent tumor tissue, and bulk CTC-enriched fractions (BCTCs) from three patients (two GBMs and one oligodendroglioma) to assess whether enriched CTCs shared tumor-specific genomic alterations with the corresponding tumors. Germline DNA from matched blood samples was also analyzed to exclude constitutional variants. Libraries were prepared according to standard protocols and sequenced on the Illumina NextSeq 2000 platform. Variants were annotated after germline subtraction, manually inspected, and compared across matched samples. Copy number analysis on tumor tissues was performed from WES data using CNVkit. Additional details on DNA extraction, library preparation, sequencing, and variant filtering are described in the Data Supplement.

### Low-Pass Whole-Genome Sequencing of Single CTCs

Recovered single CTCs underwent whole-genome amplification followed by low-pass whole-genome sequencing to assess chromosomal aneuploidies and copy number alterations (CNAs). Libraries were sequenced on the Illumina NextSeq 2000 platform, and CNA profiles were analyzed using ichorCNA and CNApp. Detailed experimental and computational procedures are reported in the Data Supplement.

### Primary GBM Cell Cultures and scRNA-Seq

Primary cell cultures were established from three *IDH1/2* wild-type GBM samples (GB58, GB127, and GB122). Tumor tissues were dissociated into single-cell suspensions and cultured under standard conditions. For single-cell RNA sequencing (scRNA-seq), viable single-cell suspensions were processed using the 10× Genomics Chromium Single Cell 3′ platform and sequenced on an Illumina NextSeq 2000 instrument. Raw data were processed in R using Seurat for quality control, normalization, dimensionality reduction, and clustering. CNAs were inferred at the single-cell level using SCEVAN to distinguish tumor from non-tumor cells. Further details on cell culture conditions, library preparation, sequencing settings, and downstream analyses are provided in the Data Supplement.

### Statistical Analysis

CTC counts were normalized to 5 mL of analyzed blood. Statistical significance was assessed using the Kruskal-Wallis test, followed by pairwise comparisons using the Dwass-Steel-Critchlow-Fligner test. Additional analytical details are reported in the Data Supplement.

The analytical workflow for CTC isolation and processing is illustrated in the Data Supplement (Fig S1)t; this section also provides further details on experimental procedures, sequencing protocols, and bioinformatic analyses.

## RESULTS

### Enriched Blood-Derived Fractions Contain CTCs

**FIG 1.**
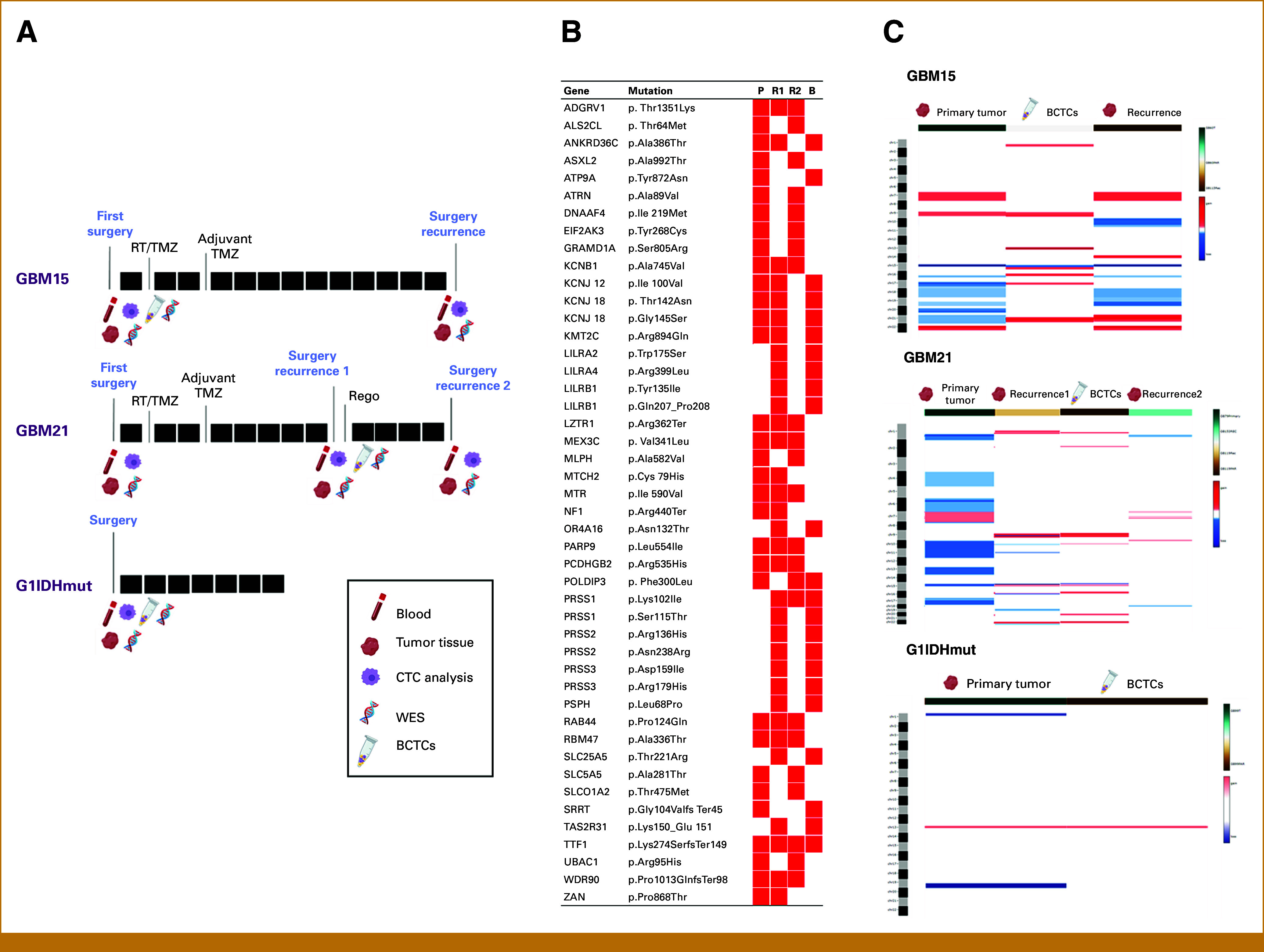
WES analysis on GBM samples: (A) Sample collection for WES from patients with GBM: Blood and tumor tissue were obtained from all patients at primary surgery and at recurrences. Blood samples were used for both CTCs analysis and BCTC isolation, and WES was performed on the BCTCs. WES was also performed in parallel on tumor tissue. Patient GBM15 contributed samples at primary surgery and one recurrence, patient GBM21 at primary surgery and two recurrences, and patient G1IDHmut at primary surgery. Symbols on the right indicate the type of material collected (blood, tumor tissue, or BCTCs) and the analyses performed (WES or CTCs analysis). (B) Scheme of patient GBM21's somatic mutations: The figure illustrates the somatic mutations detected in this patient that are shared among the primary tumor, the recurrences, and the bulk CTCs. (C) Genome-wide chromosome arm can profile heatmap for matched primary tumor, recurrences and BCTC with CNApp tool: GBM15: on the left, the CNA profiles of the primary tumor, in the middle the BCTCs and on the right the recurrence are shown; GBM21: on the left, the CNA profiles of the primary tumor, in the middle the first recurrence and the BCTCs collected at the time of the recurrence1 and on the right the second recurrence are shown; G1IDHmut: on the left, the CNA profiles of the primary tumor and on the right the BCTCs. The chromosomal amplifications are shown in red, and the deletions in blue. B, BCTCs; BCTC, bulk CTC; CNA, copy number alteration; CTC, circulating tumor cell; GBM, glioblastoma; P, primary tumor; R1, first recurrence; R2, second recurrence.

To confirm the presence of CTCs in our patients with GBM, we performed WES on BCTCs obtained after the CTCs enrichment in 2 *IDH1/2* wild-type (GBM) and 1 *IDH1*-mutated (oligodendroglioma) patients (GBM15, GBM21, and G1IDHmut). The results were compared to the mutational landscape and to the copy number genomic profile of the corresponding patients' primary tumors and recurrences. The study design for the analyses of these three patients is presented in Figure 1A.

From WES analysis for GBM15, we identified 180 variants in the primary tumor, 190 in the recurrence, and 55 in the BCTCs samples. GBM21 showed 135 variants in the primary tumor, 132 in the first recurrence, 98 in the second recurrence, and 52 in the BCTCs sample (collected during the first recurrence surgery). Finally, G1IDHmut had 109 variants in the primary tumor and 50 in the BCTCs samples.

Several somatic variants were shared across primary and recurrent tumors, considering only the coding mutations. For GBM15, 73 variants were common between the primary tumor and recurrence. When also considering the BCTCs, six variants were shared among all three samples, including three mutations in the *PRAMEF20*, one in *PRKRA*, one in *LILRA4*, and one in *KCNJ12* genes. All identified mutations for GBM15 are listed in the Data Supplement. In GBM21, some mutations were common between the primary tumor and the two recurrences, as detailed in Figure 1B; however, when including the BCTCs, one common mutation was found across all four samples, located in the *TTF1* (Fig 1B).

Finally, in the *IDH*-mutated sample, G1IDHmut, we identified four mutations shared with the BCTCs, specifically in the *HAUS1*, *HOXB7*, *MYO3A*, and *RBM12B* genes. All mutations are shown in the Data Supplement. Common mutations between BCTCs and the primary and recurrent tumors of each patient are summarized in the Data Supplement (Table S1).

Furthermore, CNA analysis was also conducted from WES data as shown in Figure 1C. These data showed a clear chromosomal alteration signature that was shared across the primary tumor, BCTC, and any recurrent samples. This highlighted how the CTCs faithfully reflected the genomic profile of the tumor.

### CTCs Counting in Patients With GBM

A total of 36 blood samples (n = 36) were analyzed for CTC enumeration from the 27 enrolled patients (n = 27), with some individuals providing multiple samples over the course of the study. The CTCs count of all 27 patients were listed in Table 2.

**TABLE 2.** Summary Table of Clinical Data and CTCs Counts for the 27 Patients (n = 27) With GBM Included in the Study

Patient	Primary Tumor	Recurrence 1	Recurrence 2
	CTC Count	CTC Count	CTC Count
GBM1	36	—	—
GBM2	—	0	—
GBM3	1	—	—
GBM4	92	—	—
GBM5	4	0	—
GBM6	—	1	—
GBM7	—	18	—
GBM8	12	—	—
GBM9	—	6	—
GBM10	11	—	—
GBM11	6	—	—
GBM12	—	8	—
GBM13	5	—	—
GBM14	0	—	—
GBM15	5	13	4
GBM16	27	3	—
GBM17	21	2	—
GBM18	33	—	—
GBM19	1	—	—
GBM20	12	3	—
GBM21	0	3	3
GBM22	11	—	—
GBM23	0	3	—
GBM24	12	—	—
GBM25	—	5	—
GBM26	11	—	—
GBM27	—	12	—

NOTE. The data include the number of CTCs detected in the primary tumor, first recurrence (recurrence 1), and, when available, second recurrence (recurrence 2).

Abbreviations: CTC, circulating tumor cell; GBM, glioblastoma.

**FIG 2.**
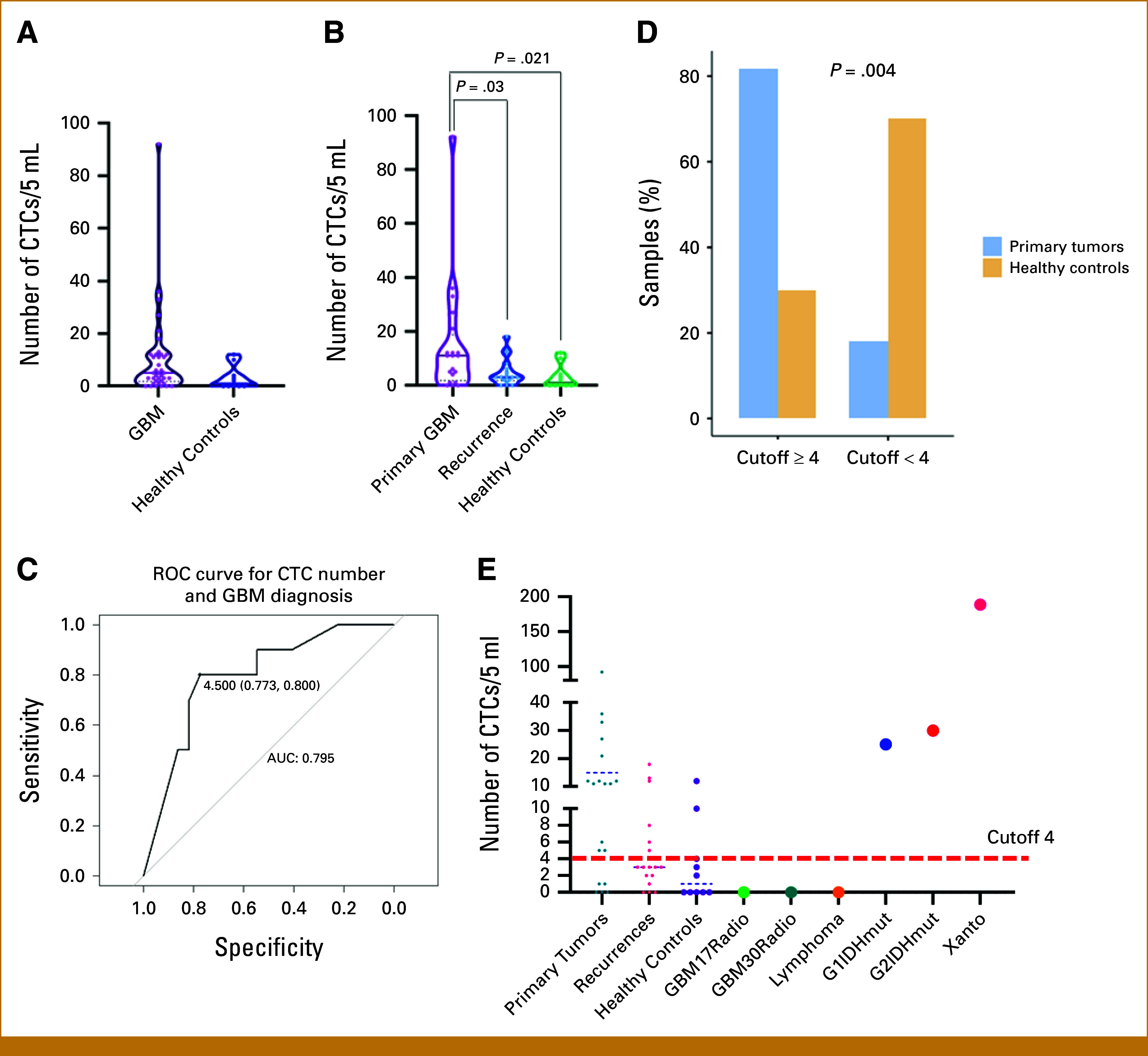
CTC counts in patients with GBM and healthy controls: (A) Distribution of CTCs detected in 5 ml of blood from patients with GBM and healthy controls, and (B) from patients with GBM subdivided into primary tumors and recurrences. (C) ROC curve analysis for CTCs counts in patients with primary GBM versus healthy controls: the ROC curve illustrates the diagnostic performance of CTC enumeration, yielding an AUC of 0.795. The optimal cutoff point identified was 4.5 CTCs. (D) Distribution of samples by cutoff value: The bar chart illustrates the percentage of primary tumor and healthy control samples based on a cutoff value of 4. The blue bars represent primary tumors, and the yellow bars represent healthy controls. A statistically significant difference (*P* = .004) is observed in the distribution between the two groups. A greater proportion of primary tumor samples (82%) fall into the cutoff ≥4 category, whereas the majority of healthy control samples (70%) are in the cutoff <4 category. (E) Distribution of CTCs counts across analyzed samples: the figure shows the distribution of CTCs counts for different sample categories. The red dashed line represents the established cutoff value. The groups include primary tumors, recurrences, healthy controls, and six nonhealthy controls (lymphoma, two radionecrosis, two oligodendrogliomas, and a xanthoastrocytoma). Each dot represents a single sample. CTC, circulating tumor cell; GBM, glioblastoma.

Additionally, for samples GBM15, GBM21, and G1IDHmut, where WES was performed on BCTCs, a matched blood sample was taken concurrently at the time of surgery to enable single CTCs counting. Among these 36 samples, we observed a greater number of GFAP-positive circulating cells in the blood of patients with GBM (median 5, range 0-92) compared with healthy controls (median 1, range 0-12) (Fig 2A). Notably, patients with primary tumors exhibited a significantly higher count (median 11, range 0-92) when compared with both healthy controls (*P* = .021) and patients with recurrence (median 3, range 0-18) (*P* = .03) (Fig 2B). It is important to consider that blood from healthy individuals normally contains small quantities of nonhematopoietic cells, including some that may resemble CTCs despite originating from nontumoral tissues. To establish a diagnostic threshold for CTC detection in primary GBM, we performed a receiver operating characteristic curve analysis to evaluate the discriminatory power of CTCs counts between patients with GBM and healthy controls (Fig 2C). The resulting AUC was 0.795, indicating good diagnostic performance. The optimal cutoff value, defined by maximizing the Youden index, was 4.5 CTCs, corresponding to a sensitivity of 80% and a specificity of 77.3%. Accordingly, we selected a threshold of 4 CTCs, considering samples with ≥4 CTCs as positive. This cutoff was applied to the CTCs counts performed in primary tumors. As shown in Figure 2D, 82% of patients with primary GBM had a CTCs count above the defined threshold, while 18% had a count below the cutoff. Conversely, in healthy controls, 70% of samples were below the cutoff, and 30% exceeded it (*P* = .004).

### CTCs as a Potential Tool to Distinguish Glioma Recurrence From Radionecrosis and Nonglioma Brain Lesions

The analysis of 6 non-GBM controls (n = 6) supported the cutoff's validity. Radionecrosis and lymphoma samples consistently yielded 0 CTCs. Conversely, both IDH-mutant gliomas and pleomorphic xanthoastrocytoma (established brain tumors) displayed CTCs counts exceeding the threshold. These preliminary observations suggest that CTCs enumeration may assist in distinguishing neoplastic from non-neoplastic brain lesions, such as radionecrosis. The clinical details and CTCs counting of the six unhealthy control patients were listed in the Data Supplement (Table S2). The data shown in Figure 2E corroborate our previous findings and support the validity of the chosen cutoff.

### Single CTCs Molecular Characterization Through CNAs Analysis

**FIG 3.**
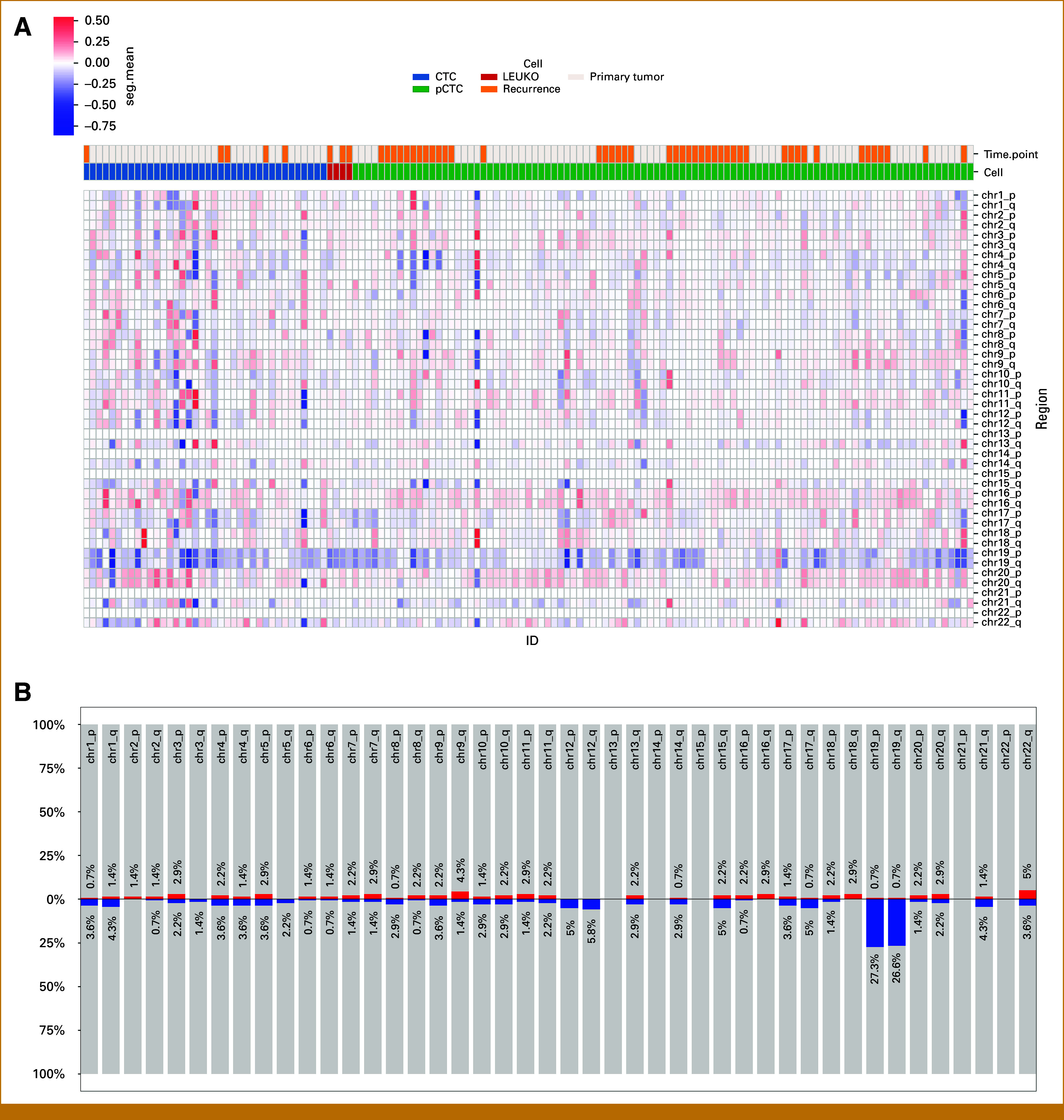
CNA profiles of isolated CTCs. (A) This heatmap displays the CNA profiles for each analyzed CTCs. Columns represent individual CTCs, while rows correspond to the chromosomal arms (p and q) for chromosomes 1 through 22. The color scale indicates the type of copy number alteration: red signifies amplification and blue signifies deletion, with color intensity reflecting the magnitude of the alteration (see the legend on the left). The bars at the top provide additional information about each sample. The cell type row distinguishes between the different cell types analyzed: CTCs, pCTCs, and leukocytes (see the legend). The time point row categorizes samples based on when they were collected: primary tumor (gray) and recurrence (yellow). It is important to note that the deletions observed on chromosome 19 are considered technical artifacts and do not represent true biologic alterations. (B) Frequency of CNAs across CTCs. This Region Frequencies plot displays the percentage of recurring CNAs across all analyzed CTCs. The x-axis represents the chromosomes (chr1 to chr22). The stacked bars for each chromosome show the frequency of different CNA events. Red segments indicate the percentage of samples with an amplification in that region, whereas blue segments show the percentage of samples with a deletion. Please note that the high frequency of deletions on chromosome 19 is considered a technical artifact. CNA, copy number alteration; CTC, circulating tumor cell; pCTC, putative CTC.

CTCs were isolated using the DEPArray NxT system from 24 samples (n = 24). Given the absence of a universally accepted single marker for GBM tumor cells, a population termed putative CTCs (pCTCs) was also evaluated. These pCTCs were identified by Hoechst staining for nucleus and morphological characteristics consistent with CTCs, representing a broader assessment of circulating populations potentially originating from the tumor. Figure 3A illustrates the diverse chromosomal alterations observed in individual CTCs and pCTCs from our patient cohort. Among the CTCs, we observed significant heterogeneity in alterations and identified cells lacking detectable CNAs. Figure 3B shows the overall genomic instability and the most frequently altered chromosomal segments within all samples.

Aggregating all analyzed CTCs, we observed that 51% of CTCs from primary tumors exhibited CNAs, while 61% of CTCs from recurrent tumors exhibited CNAs. The observation of CTCs lacking chromosomal alterations is a potentially innovative finding, suggesting that these circulating cells may originate directly from the TME rather than being exclusively malignant tumor cells.

### Single Cell RNA Seq on GBM Primary Cancer Cell Lines

**FIG 4.**
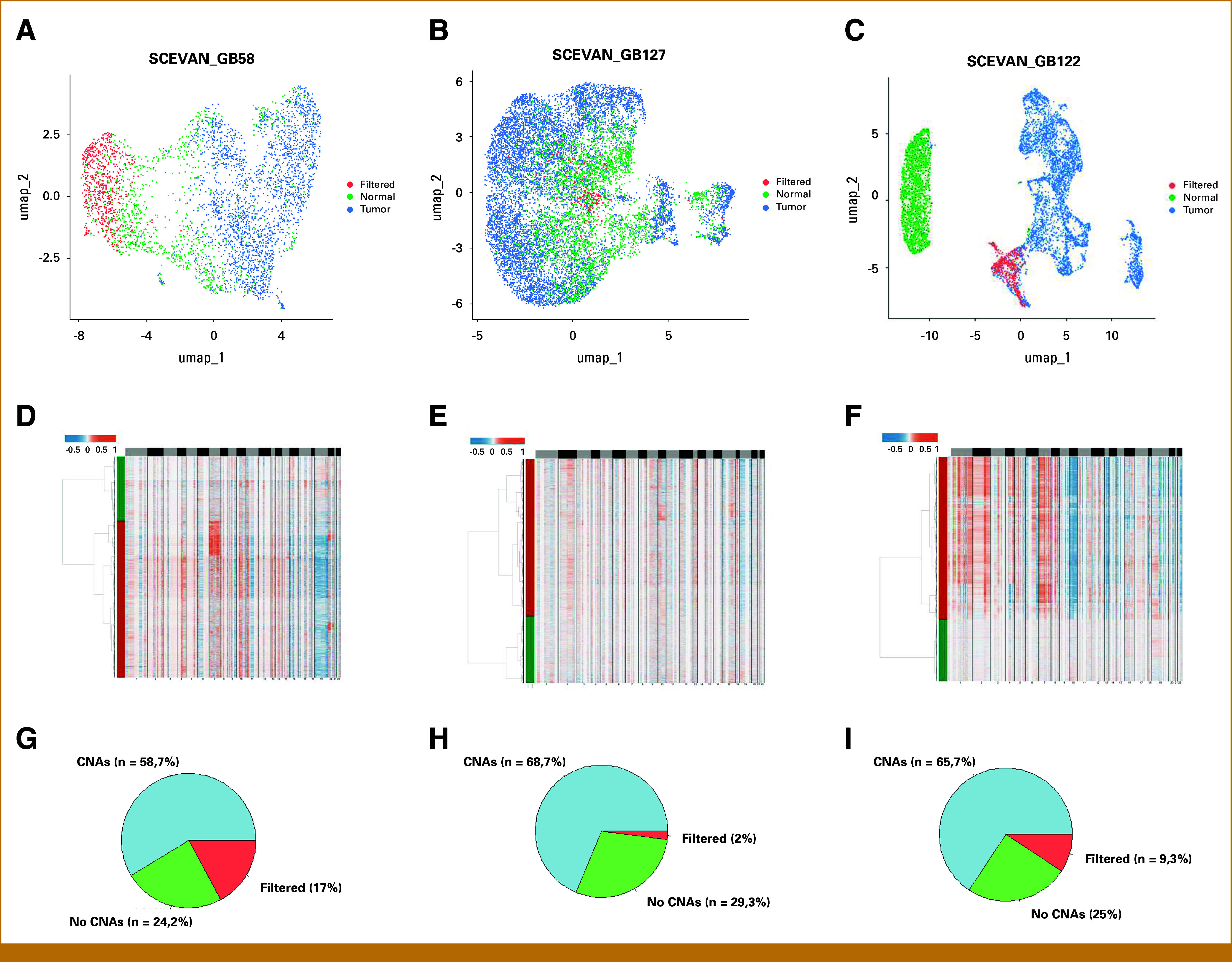
SCEVAN software analysis of scRNAseq data from three primary GBM cell lines for CNAs identification. (A-C) UMAP plot of GB58, GB127, and GB122 primary cell lines, tumor refers to cells with CNAs, whereas normal refers to cells without CNAs. (D-F) Heatmaps illustrating the landscape of chromosomal alterations, indicated as gain (red) and loss (blue). On the left, cells lacking alterations are identified in green, whereas cells with chromosomal alterations are in red. (G-I) Percentage of cells with CNAs (light blue) and without CNAs (green). Cells called filtered are not analyzable, shown in red. CNA, copy number alteration; GBM, glioblastoma; UMAP, Uniform Manifold Approximation and Projection.

To better investigate the cellular composition of GBM, single cell RNA Seq (scRNA-seq) analysis was performed on three primary GBM cell lines (GBM58, GBM127, and GBM122). In Figures 4A‐4C, the Uniform Manifold Approximation and Projection plot shows the clustering of cells based on their CNA profiles. Here, each cell cluster is categorized by its CNAs profile, designating it as either having CNAs (CNAs) or lacking CNAs (NO CNAs). Cells that the software could not confidently classify were excluded from the plot (filtered). In Figures 4D‐4F, heatmaps illustrate the landscape of chromosomal alterations, with unaltered cells shown in green and altered cells in red. Crucially, our analysis of these primary GBM cell lines also identified the presence of cells lacking chromosomal alterations, ranging from 24% to 29% (Figs 4G-4I). This finding is analogous to the results obtained from the CTCs analysis, although with a slightly lower proportion of CNAs-negative cells in the cell lines compared to the circulating population.

## DISCUSSION

GBM remains one of the most therapeutically challenging malignancies, defined by its heterogeneity and inevitable recurrence.^15^ Although extracranial metastasis is exceedingly rare in GBM, the detection of CTCs in affected patients observed in multiple studies^8-10,16^ challenges the belief that GBM is strictly a disease localized to the CNS and unable to cross the blood-brain barrier. Furthermore, a 2014 study by Jimsheleishvili et al^17^ revealed that multiple solid organ transplant recipients contracted extracranial GBM after receiving organs from patients with GBM, which strongly supports the existence of CTCs.

A primary objective was confirming the neoplastic origin of isolated CTCs. Our data show that BCTCs recapitulate the primary tumor's genetic features. By sequencing BCTCs instead of using only single-cell approaches, we captured a broader mutational spectrum, including subclonal alterations associated with disease progression and recurrence.

Previous studies in other tumor types^18,19^ have reported bulk CTCs sequencing; however, these analyses almost invariably required whole genome amplification (WGA) to compensate for the limited DNA yield, a process known to introduce amplification bias and artifactual variants. In contrast, our workflow achieved bulk CTCs sequencing without WGA, thereby preserving the native genomic profile of the captured cells and minimizing potential artifacts. To our knowledge, this is the first study to assess the genomic composition of CTC-enriched samples specifically in GBM and the only one to do so without the confounding effects of WGA.

In our cohort, several shared mutations were identified between BCTCs and their corresponding primary or recurrent GBM tissues. Among these, we highlight genes with reported or potential relevance to GBM biology.^20-22^

Numerous studies across various cancer types have highlighted the clinical utility of CTCs counts, often associating them with prognosis, progression-free survival (PFS), and OS.^23-27^ Our study extends this evidence to GBM, where we established a clinically relevant cutoff of four CTCs, derived from healthy controls that effectively discriminated primary GBM cases. Importantly, GFAP, although a well-established astrocytic marker, can also be expressed in other somatic cell types such as fibroblasts, chondrocytes, myoepithelial cells, and stellate cells^28^ underscoring the need for a diagnostic threshold to distinguish pathologic from physiologic circulating cells. Applying this threshold, 70% of patients with primary GBM exceeded the cutoff compared with only 20% of healthy controls, whereas patients with recurrent GBM showed a markedly lower proportion (33%), suggesting distinct biologic dynamics between primary and recurrent disease. To date, no studies have defined a quantitative cutoff for CTCs enumeration in GBM, and our investigation represents the first attempt to propose such a threshold within this disease context. In contrast, several malignancies have well-established CTCs cutoffs, which have been validated as prognostic biomarkers. For instance, a threshold of ≥5 CTCs per 7.5 mL of blood has been consistently applied in metastatic breast^29^ and prostate cancers,^30^ while a cutoff of ≥3 CTCs per 7.5 mL has been reported for colorectal cancer.^31^ The 4 CTC cutoff was applied exclusively to primary GBM, as the clinical history of recurrent cases—marked by prior surgery and adjuvant therapies—makes comparison with healthy controls unreliable. Preliminary data suggest that radionecrosis may be a more appropriate reference for recurrent disease. The lower CTC counts in recurrence may reflect earlier detection and management compared with the potentially longer shedding window in primary GBM. Non-GBM cases (radionecrosis and lymphoma) lacked CTCs, reinforcing the cutoff's validity for differential diagnosis, although these results remain preliminary because of the small cohort. Although current sample sizes preclude robust correlations with PFS or OS, these descriptive biologic observations underscore the need for larger prospective studies to define the prognostic and clinical utility of CTC enumeration in GBM.

A key part of our study involved the molecular characterization of CTCs, focusing on CNAs. Advancing beyond bulk analysis, the single-cell resolution achieved via DEPArray NxT isolation and low-pass whole-genome sequencing was critical. This approach enabled precise assessment of genomic alterations, which serve as robust indicators of genomic instability and clonal evolution. We observed substantial heterogeneity in CNA profiles among individual CTCs, including the consistent detection of cells lacking detectable CNAs. Although the presence of CNAs in circulating cells reinforces their malignant nature,^32^ the occurrence of CTCs without chromosomal alterations is an unexpected and potentially innovative observation. This raises the intriguing hypothesis that these circulating cells may originate directly from the TME. They could be stromal or immune cells that cocirculate with malignant CTCs, rather than being tumor cells themselves. These finding challenges conventional views and expands the potential information derivable from liquid biopsies, offering insights into the complex cellular ecosystem of the tumor and its interplay with the circulation. Using the SCEVAN software to infer CNAs from scRNAseq data on GB58, GB122, and GB127 cell lines, we found that between 30% and 50% of the cells in each GBM line were wild type from a CNA point of view, consistent with our findings from the CTC analysis. These results further support the hypothesis that the presence of CTCs lacking CNAs reflects the intrinsic heterogeneity of GBM. This heterogeneity, inherent to the tumor, is clearly mirrored in the circulating compartment. The idea that CNA-negative CTCs may originate from the TME aligns with the current understanding of GBM biology. The tumor mass comprises various cell types, including reactive glial cells (GFAP positive and mitotically active), which can be morphologically indistinguishable from tumor cells. This phenomenon has long been recognized in the field.^33^ Given the variability in GFAP expression, where more aggressive and undifferentiated tumor cells tend to be GFAP negative, and more differentiated or mature cells are GFAP positive,^34^ it is plausible that some rare circulating cells, even in the absence of typical malignant CNAs, are still part of the broader tumor ecosystem. This suggests that CTC studies may capture not only genetically altered, highly anaplastic tumor cells but also other cellular players involved in tumor dynamics and progression.

In conclusion, this study demonstrates that CTCs are consistently detectable in patients with GBM and provide real-time genomic information. Bulk analysis enabled the detection of clinically relevant mutations, while single-cell profiling revealed the extent of molecular heterogeneity. Notably, we also identified a subset of CNA-negative CTCs, potentially originating from the TME. Despite limitations in sample size and single-cell yield, these findings support the value of CTCs as a liquid biopsy tool for disease monitoring, recurrence assessment, and personalized treatment because they offer a more versatile approach that can be employed from the outset of the disease course, irrespective of a priori knowledge of a specific molecular target. Future studies should refine CTC isolation and functionally characterize distinct subpopulations to clarify their biologic roles.
